# Effects of Low-Temperature Heat Treatment on Mechanical and Thermophysical Properties of Cu-10Sn Alloys Fabricated by Laser Powder Bed Fusion

**DOI:** 10.3390/ma17122943

**Published:** 2024-06-15

**Authors:** Edem Honu, Selami Emanet, Yehong Chen, Congyuan Zeng, Patrick Mensah

**Affiliations:** 1 Department of Mechanical Engineering, Southern University and A & M College, Baton Rouge, LA 70807, USA; edem.honu@sus.edu (E.H.); patrick_mensah@subr.edu (P.M.); 2 Department of Mechanical & Industrial Engineering, Louisiana State University, Baton Rouge, LA 70803, USA; semane1@lsu.edu (S.E.); yche136@lsu.edu (Y.C.)

**Keywords:** Cu-10Sn, metal additive manufacturing, heat treatment, thermal diffusivity, hardness

## Abstract

This study investigated the impact of low-temperature heat treatments on the mechanical and thermophysical properties of Cu-10Sn alloys fabricated by a laser powder bed fusion (LPBF) additive manufacturing (AM) process. The microstructure, phase structure, and mechanical and thermal properties of the LPBF Cu-10Sn samples were comparatively investigated under both the as-fabricated (AF) condition and after low-temperature heat treatments at 140, 180, 220, 260, and 300 °C. The results showed that the low-temperature heat treatments did not significantly affect the phase and grain structures of the Cu-10Sn alloys. Both pre- and post-treatment samples displayed consistent grain sizes, with no obvious X-ray diffraction angle shift for the α phase, indicating that atom diffusion of the Sn element is beyond the detection resolution of X-ray diffractometers (XRD). However, the 180 °C heat-treated sample exhibited the highest hardness, while the AF samples had the lowest hardness, which was most likely due to the generation of precipitates according to thermodynamics modeling. Heat-treated samples also displayed higher thermal diffusivity values than their AF counterpart. The AF sample had the longest lifetime of ~0.19 nanoseconds (ns) in the positron annihilation lifetime spectroscopy (PALS) test, indicating the presence of the most atomic-level defects.

## 1. Introduction

Cu-Sn (tin bronze) is a copper-based alloy composed of copper (Cu) as the base metal and tin (Sn) (ranging from 5 to 25 wt.%) as its primary alloying element [1]. Tin bronze possesses outstanding mechanical and thermophysical properties, such as high strength, excellent ductility, good corrosion resistance, and good electrical conductivity [2,3,4]. Cu-10Sn alloy has been extensively explored in bearing applications, hydraulic fittings, pump linings, utensils, bearings, sheets, rods, and wires owing to its excellent hardness, yield strength, wear resistance, good corrosion resistance, and high thermal conductivity [1,4,5,6,7].

Conventional methods such as wrought, casting, forging, extrusion, and metallic alloying have been extensively explored in the past for the fabrication of Cu-10Sn alloys and other metallic alloy parts [8,9,10,11]. However, with recent advances, the laser powder bed fusion (LPBF) additive manufacturing (AM) process has also gained significant attention as a viable rapid metal parts fabrication process to supplant conventional fabrication processes and achieve similar desired mechanical properties with complex shapes using lower production lead times and minimal material waste [2,12,13,14,15]. Additionally, it has also been considered extensively for research and development of advanced engineering materials because of its higher cooling rates, higher fabrication resolutions, and better surface finishes compared with other additive manufacturing processes, such as laser powder direct energy deposition (LP-DED) [16]. The LPBF process employs a focused laser beam to melt and fuse metallic powders, creating complex shaped parts with almost full density that cannot be achieved using conventional fabrication methods [5,13,17].

The fabrication of Cu-10Sn alloy parts using the LPBF process has also gained significant interest among designers seeking to advance the fabrication process and optimize the microstructural, mechanical, thermophysical, and corrosion-resistant properties of the as-built alloys and tailor them for specific applications [2,5,18]. However, presently, the primary challenge towards the advancement of the LPBF process (or even metal AM in general) for parts fabrication is establishing a better understanding of the correlation between process parameters, microstructural properties, and final built material properties [15,19,20]. Notwithstanding, regardless of the process parameters used in the LPBF process, there are typically significant residual stresses induced by the high cooling rates and temperature gradient of the AM process which can markedly influence crack propagation and fatigue properties [21]. Several post-heat treatment techniques of LPBF parts have been explored extensively to alleviate such thermal/residual stresses and optimize the microstructural and mechanical properties of the final build for specified applications [22]. These post-processing steps have been employed as efforts are still being made to establish standard process parameters and material property relationships towards achieving high quality LPBF parts with less defects.

Generally, heat treatment techniques involve a well-controlled heating and cooling process used to alter the microstructure of the as-built metallic parts, consequently improving the mechanical and physical properties [23]. Although the use of these standard post-heat treatment techniques (developed specifically for traditional manufacturing) for AM parts has come under extreme scrutiny and has raised many concerns, efforts are continually being made to evaluate their applicability for metal AM parts to achieve desired properties [22]. Typical post-heat treatment techniques applied to fabricated metals and metallic alloys noted in the literature can be classified as solution treatment [24], stress relieving [25], and hot isostatic pressing (HIP) [26]. In addition, alternative techniques such as vacuum annealing (VA) tailored to specific alloy groups with diverse melting points and treatment temperature ranges are also available [5,27,28]. However, such heat treatments are typically conducted at high temperatures compared with the melting points of the alloys, which significantly affect the grain structures of the LPBF samples. Due to these high temperatures, recrystallization and grain growth typically occur (grain sizes become larger compared with those of as-fabricated (AF) parts), which significantly alters the grain structures of AF LPBF parts, consequently affecting the properties of the additively manufactured parts [5,29,30]. Specifically, it was reported that a grown grain size would lead to a decrease in mechanical strength of the parts due to the Hall–Petch effect. In addition to the mechanical and microstructural characterization work performed [2,18], thermophysical and corrosion performance of Cu-10Sn alloys fabricated using the LPBF-AM process has also gained a lot of interest [5]. Thermophysical properties, i.e., thermal conductivity and thermal diffusivity, are well known to be significantly affected by atomic-level defects, including point defects such as vacancies, solid solution atoms, and line defects like dislocations [31]. Due to the extremely fast cooling rates and significant thermal gradients of the LPBF process, dense vacancies and dislocations typically exist in the LPBF parts [32,33], which are obviously heat transfer barriers.

Since L-PBF Cu-10Sn alloy parts typically tend to also contain process-induced defects such as point defects and dislocations caused by rapid cooling process, Zeng et al.’s [5] prior research essentially sought to minimize these defects through high-temperature post-heat treatment methods. They explored a VA heat treatment technique and compared the microstructural, mechanical, and thermophysical properties of the AF samples and VA samples. However, it was reported that the VA samples exhibited lower mechanical and thermophysical properties than the AF samples due to the significantly enlarged grain sizes and the formation of solid solutions by the dissolving of the Sn-rich phase into the Cu matrix (greatly increased solid solute atoms) after VA treatments.

As discussed above, LPBF samples typically have high residual stresses, including point defects and dislocations within the materials [34]. These are deemed barriers to thermal conductivity [31]. Based on this, researching methods to reduce the concentration of defects, such as vacancies, solid solute atoms, and dislocations, to improve thermal conductivity while maintaining mechanical performance without affecting the grain structures (maintain the Hall–Petch effects) is worthwhile. Thus, to address the gaps noted regarding existing high-temperature post-heat treatments, for example the VA post-heat treatments of LPBF Cu-10Sn alloys conducted by Zeng et al. [5], the present study aimed to minimize the LPBF-AM process-induced defects without significantly altering the grain structures using an unconventional heat treatment technique.

Thus, a novel low-temperature heat treatment routine (with heat treatment temperatures much lower than traditional methods) was explored. Specifically, heat treatment temperatures of 140, 180, 220, 260, and 300 °C were evaluated based on the typically recommended stress-relieving heat treatment temperatures of 190–288 °C [35]. The present study expanded the temperature range to gain a better understanding of the effect of temperature on the performance of the Cu-10Sn alloy. Subsequently, the evolution of atomic-level defects and microstructural, phase structural, thermophysical, and mechanical investigations were carried out to evaluate the impacts of low-temperature heat treatments on the mechanical and thermophysical properties of the LPBF Cu-10Sn alloys. Thermodynamic calculation of phase diagrams was then employed to verify hardness value results and speculations regarding the evolution of the mechanical performance of LPBF Cu-10Sn samples.

## 2. Materials and Methods

### 2.1. Specimen Preparation

In this study, spherical Cu-10Sn alloy powders (with d10, d50, and d90 to be 15.5 μm, 25.9 μm, and 37.6 μm, respectively) obtained from Concept-Laser GmbH were used for LPBF fabrication using the Concept-Laser Mlab cusing R LPBF system under pure inert argon gas conditions. The standard LPBF processing parameters, specific to the LPBF system employed for sample fabrication, are listed in Table 1. In addition, the island scanning strategy was employed using a square island size of 5 × 5 mm. The island scanning strategy was chosen against the raster scanning strategy because this scanning strategy has been noted for improving isotropic properties and reducing the texture index of LPBF-AM parts [36,37]. Details showing the morphology of the Cu-10Sn powders, the fabrication process, and image illustrations of AF samples built under two orientations (vertical and horizontal) can be found in Zeng et al.’s previous study [5].

It is noteworthy to point out that Zeng et al.’s prior work also evaluated the effect of building direction on the evolution of mechanical and thermophysical properties of the AF samples. They reported that the AF samples prepared using the two building orientations showed similar compression strengths and thermal conductivities. This clearly indicated that the building orientation had no significant effect on the microstructural evolution and properties of the AF samples. Thus, for the present study, only the horizontal-oriented AF samples were investigated.

To investigate the impact of low-temperature heat treatment on the LPBF samples, Cu-10Sn samples (wrapped with stainless steel foil to minimize oxidation) were heated up to (10 °C/min) and kept at 140, 180, 220, 260, and 300 °C for 60 min, then were fast-cooled by water quenching (all the processes were under air atmosphere). In the remainder of this study, samples under investigation were denoted simply based on heat treatment conditions for easy distinguishing, namely AF, 140 °C, 180 °C, 220 °C, 260 °C, and 300 °C.

### 2.2. Testing Procedures

Cylindrical rod specimens measuring 14.0 mm in diameter and 15.0 mm in height, oriented horizontally, were fabricated and evaluated. Thermal diffusivity assessments were conducted using a state-of-the-art Netzsch LFA 467 HT HyperFlash^®^-light flash instrument (NETZSCH Corp., Selb, Bayern, Germany) from room temperature to 250 °C, with a temperature interval of 50 °C. For thermal diffusivity measurements, thin disk-shaped samples with dimensions of Φ12.56 × 2.55 mm were cut from the cylindrical samples using a wire electrical discharge machining (EDM) system. The cutting was performed such that the top and bottom surfaces of the disks were perpendicular to the central axis of the cylinders. Subsequently, the top and bottom surfaces of the disks were ground using SiC papers with grit sizes of 320 and 600, successively. Following the ethanol cleaning process, both the reference sample (a pure copper material sourced from NETZSCH Corp., Selb, Bayern, Germany) and each target sample were coated with a thin uniform layer of graphite to ensure consistent heat absorption. The thermal diffusivity (α) was then directly measured using the LFA 467 apparatus. For the repeatability and credibility of the test results, three samples at each condition were tested. Additional information and a comprehensive description of the thermophysical testing procedure can be found in the previous research conducted by Zeng et al. [5].

A hardness test was also performed for both AF samples and samples subjected to low-temperature heat treatments. Prior to the test, the top surface (vertical to the central axis of the cylinders) of each hardness testing sample was mechanically ground to a mirror finish, using SiC grinding papers of 320, 600, 800, 1000, and 1200 grits, sequentially. More than five spots were tested for each sample condition (AF and heat treatment condition) using the Vickers hardness tester at room temperature with a test load of 500 gf. Further details regarding the protocol for the Vickers hardness testing can be found in the provided references [38,39].

### 2.3. Material Characterization

**Figure 1:**
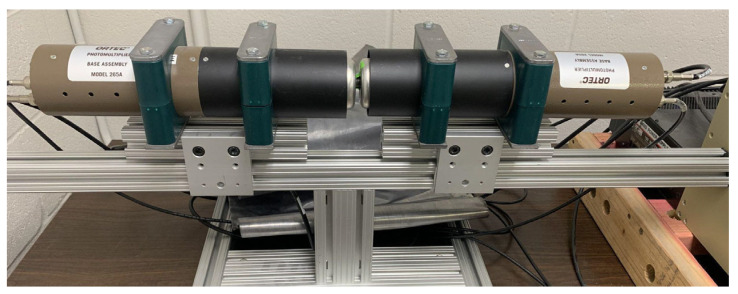
PALS experimental setup showing the photomultiplier base assemblies for atomic level defect detection.

To evaluate the relative content of atomic-level defects in the AF samples and the heat-treated samples, positron annihilation lifetime spectroscopy (PALS) analysis, an Ortec^®^ 269 Photomultiplier Base Assembly with a sodium (^22^Na_11_) radioactive positron (antielectron) source, was utilized (ORTEC AMETEK, Oak Ridge, TN, USA). To perform the test, the ^22^Na_11_ positron source was sandwiched between the surfaces of two samples in the same condition (either in the AF condition or heat-treated conditions). The two samples were subsequently also fixed in position by the two photomultiplier base assemblies or tubes. The whole setup was embedded in aluminum foil to serve as a shield against radiation from the radioactive source. Further details and the literature on the protocol for PALS analysis for atomic defect detection can be found elsewhere [40,41]. Figure 1 depicts the laboratory setup for PALS experimental tests.

In a material, positrons are highly sensitive to areas with a high concentration of atomic-level defects or open-volume defects such as vacancies and dislocations. Upon diffusion into the material, when a positron encounters a defect site and becomes trapped, a strong attractive potential is induced and built up. The reason for the induced strong potential at the defect site is because of the absence of a repulsive positively charged nucleus that is able to repel the positron particles [40,42,43], consequently yielding a longer lifetime for the positrons.

Phase structure analysis was carried out using X-ray diffraction (XRD) on both AF and heat-treated samples. A total of θ–2θ scans were performed within the angular range of 20–100°, employing a scanning step size of 0.026°.

Microstructural examination of both AF and heat-treated samples was conducted using an FEI Quanta3D FEG dual-beam scanning electron microscope/focused ion beam (SEM/FIB) instrument equipped with an electron backscatter diffraction (EBSD) attachment. Before EBSD examinations, samples underwent mechanical grinding using SiC papers of varying grit sizes (320, 600, 800, 1000, and 1200 grits, sequentially), followed by polishing with MetaDi™ supreme polycrystalline diamond suspension (6 μm, 3 μm, 1 μm, sequentially) (PACE Technologies, Tucson, AZ, USA), and final vibratory polishing with 50 nm silica suspension on a Pace Technologies GIGA 0900 vibratory polisher (PACE Technologies, Tucson, AZ, USA) for 12 h. Then, EBSD mapping was carried out at 30 kV and 23 nA. Subsequently, the raw EBSD data were analyzed using TSL OIM software (version 7.3.1) to determine the grain sizes of the samples.

### 2.4. Thermodynamic Modeling

To confirm mechanical and thermophysical performance test results, thermodynamic calculations (CALPHAD method) were carried out using Thermo-Calc™ 2023b software and the TCCU3 (Cu alloys ver. 3) database. For the present study, the system was configured to simulate the amount of phases present at equilibrium across a temperature range (0–1500 °C) using both single point and one axis configuration for Cu-10Sn alloys. The composition of the Cu-10Sn alloy was defined in the system definer as 91.2 wt% Cu and 8.8 wt% Sn [5]. Comprehensive details and further instructions regarding Calphad equilibrium phase simulations can be found elsewhere [44,45,46].

## 3. Results and Discussion

### 3.1. Microstructural Characterization

**Figure 2:**
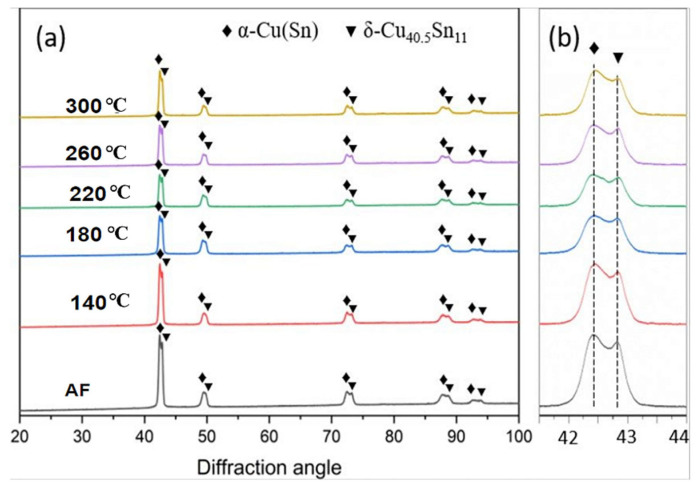
XRD results: (**a**) θ/2θ diffraction patterns from the AF and heat-treated samples over the 2θ range of 20–100°; (**b**): θ/2θ diffraction patterns displayed over a narrower 2θ range of 41–44°.

Figure 2 displays XRD θ/2θ scans obtained from AF samples and low-temperature heat-treated samples. Both the AF Cu-10Sn samples and the low-temperature heat-treated samples consisted of two phases, identified as the α-Cu(Sn) phase (Cu-rich phase) and the intermetallic compound δ-Cu40.5Sn11 phase (Sn-rich phase), indicating that Cu and Sn were not homogeneously mixed. With careful observation, the diffraction peaks for both the AF sample and its heat-treated counterparts nearly perfectly overlapped, showing no visible diffraction angle shift. Specifically, as shown in Figure 2b, the diffraction peaks for α-phase (42.5°) and the δ-phase (42.8°) remained almost constant also for both AF and all heat-treated samples. This indicates that, after the low-temperature heat treatments, no visible phase transformation occurred up to a heat-treatment temperature of 300 °C. This also reveals the fact that atom diffusion for Sn element was beyond the detection resolution of the X-ray diffractometer (XRD), unlike the dissolving process of Sn atoms into the Cu matrix through the VA process at temperatures above 600 °C [5]. In addition, the identification of both α and δ phases in all samples showed strong agreement with other prior phase structural studies by Scudino et al. [12] and Mao et al. [18] of Cu-10Sn alloy fabricated using the LPBF-AM process.

However, Mehta et al. [2] also produced LPBF Cu-10Sn samples using a laser power of 350 W and a scan speed of 750 mm/s and reported that the LPBF Cu-10Sn alloy consisted of two main phases, namely the α (Cu) solid solution and the δ phase (Cu41Sn11) phase, similar to phase constituents of the present study. However, they observed from the XRD phase analysis that the α-phase consisted of two subphases, α1 and α2, occurring at two high-intensity diffraction peaks of 42.8° and 42.5°, respectively. The δ phase was also present at the 42.7° diffraction peak but at a lower intensity. The lower fraction or intensity of the δ phase in the as-built Cu-10Sn alloys can be attributed to the phase constituents of the raw powders and also the rapid cooling of the LPBF process, which resulted in more Sn saturation in the α (Cu) phase in the as-built alloy.

Meanwhile, Wang et al. [47] manufactured Cu-15Ni-8Sn alloy using selective laser melting (SLM) with a laser power of 300–450 W and scanning speed of 500–2700 mm/s and reported that detection of the α phase in the AF alloy at high-intensity diffraction peaks between 43^o^ and 44^o^, comparable to the present study. However, interestingly, no δ phase was detected in the SLM-manufactured alloy across all diffraction peaks. They attributed the lack of detection of the δ phase to the increased solubility of Sn in the Cu-15Ni-8Sn alloy caused by the rapid cooling rate of the SLM process.

Clearly, the LPBF processing parameters used for fabrication and their corresponding cooling rates can highly influence the evolution of the phase constituents of the as-built alloy.

**Figure 3:**
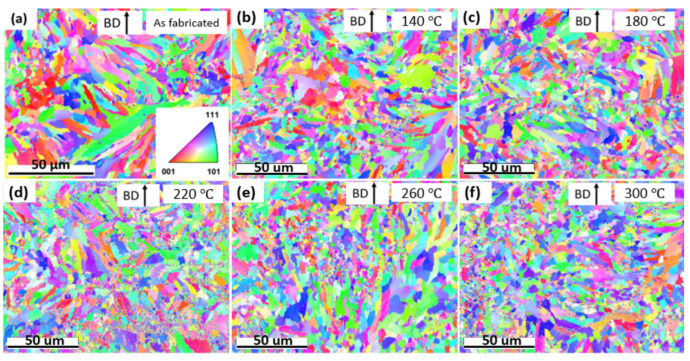
Typical EBSD IPF maps of LPBF-AM Cu-10Sn samples in horizontal orientation: (**a**) AF; (**b**) 140 °C; (**c**) 180 °C; (**d**) 220 °C; (**e**) 260 °C; and (**f**) 300 °C. Colors and their intensities represent the orientations of the grain structures, as shown in bottom right of (**a**). The upward arrows represent the building direction (BD) of the samples.

Figure 3 illustrates the EBSD inverse pole figure (IPF) orientation maps of AF and low-temperature heat-treated samples at 140 °C, 180 °C, 220 °C, 260 °C, and 300 °C. All test sample surfaces were vertical to the central axis of the cylinders and were parallel to the building direction (BD). The grain structures for the AF sample are shown in Figure 3a, with both elongated and equiaxed grains observed. Upon closer examination of Figure 3a, it becomes apparent that the elongated grains did not strictly align along the building direction. This deviation can be attributed to the complex thermal gradients resulting from the zigzag laser scanning directions and island laser scanning strategy utilized in this study. This observation is consistent with the microstructure of high strength aluminum alloy (AlSi10Mg) parts made using LPBF reported by Sidot et al. [48].

TSL OIM software was employed to estimate the average grain size using the linear interception method. For the AF sample, the value of the average grain size, d, was ~2 μm. The smaller grain size of the AF LPBF sample compared with traditional cast counterparts was attributed to the high cooling rate associated with the LPBF process [5,16].

Figure 3b–f summarize the EBSD IPF orientation maps of the low-temperature heat-treated samples. Closer observation reveals no visible grain size change after heat treatment at all heat-treatment temperatures up to 300 °C. Specifically, the average grain size for the heat-treated samples was approximately 1.9 um. In addition, clearly, grain morphologies did not change significantly after the heat treatments in this study, and both elongated and equiaxed grains existed. Following low-temperature heat treatment, the Cu-10Sn grains did not exhibit the formation of enlarged equiaxed and twinning grains, unlike what was observed in the annealed samples previously reported [5].

### 3.2. Mechanical Performance: Hardness

**Figure 4:**
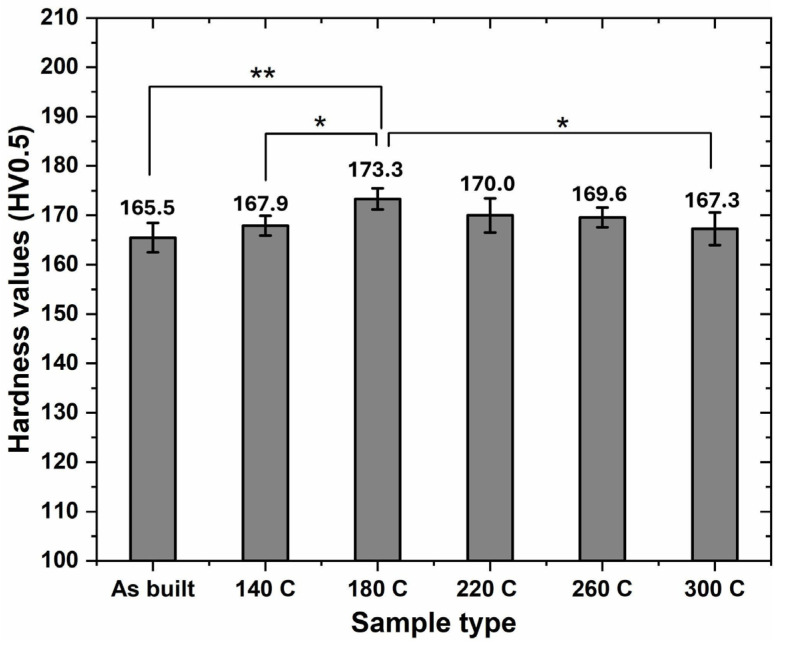
Image showing the hardness test results for AF and heat-treated Cu-10Sn alloy samples. *p* < 0.05 is strategically significant and is denoted by *, *p* < 0.01 is highly strategically significant and is denoted by **.

Figure 4 summarizes the Vickers hardness values for AF and low-temperature heat-treated samples. According to the figure, it is seen that the average hardness values of the heat-treated samples were higher than that of the AF sample. For example, the 140 °C sample exhibited a slightly higher average hardness value of ~167.9 HV0.5 than the AF sample. With careful observation, the sample heat-treated at 180 °C showed the highest average hardness value of ~173.3 HV0.5. However, as the heat treatment temperature increased over 180 °C, generally, Vickers hardness of the samples decreased. GraphPad Prism software (version 9.5.0) was used to statistically compare the hardness values of the samples, and it was discovered that the hardness of the sample heat treated at 180 °C was significantly higher than that of the samples heat treated at 140 °C and 300 °C, and highly significantly higher than that of the AF sample. Inspiringly, a low heat-treatment temperature of 180 °C can significantly improve the mechanical performance of the Cu-10Sn alloy sample made with the L-PBF process, providing a low-cost energy-saving strategy to optimize additively manufactured parts.

**Figure 5:**
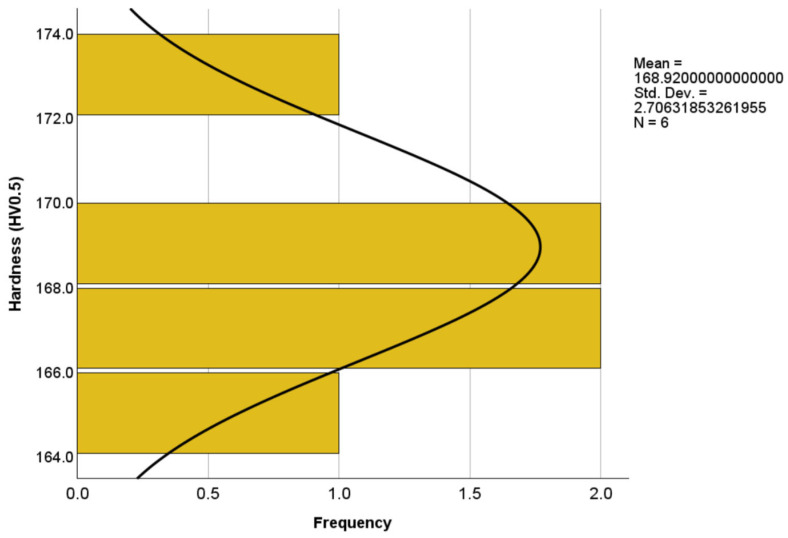
A slight positively skewed distribution of hardness value among tested samples.

A statistical analysis was conducted on the evolution of the hardness values of the samples using IBM SPSS Statistics 29.0.2.0 software to gain further insights into the validity of the data. The analysis included measures of central tendency, dispersion, and skewness of the distribution. The results showed a mean hardness value of 168.92 HV0.5 with a standard deviation (SD) of 2.71. Observing Figure 5, the distribution of the hardness values appeared to be almost symmetric around the mean, suggesting a roughly normal distribution. However, a closer examination reveals a slight positive skewness, indicating a minor asymmetry with a longer tail on the right side. This slight skewness was primarily attributed to the peak hardness value observed for the heat-treated sample at 180 °C, which acted as an outlier in the hardness value results.

**Figure 6:**
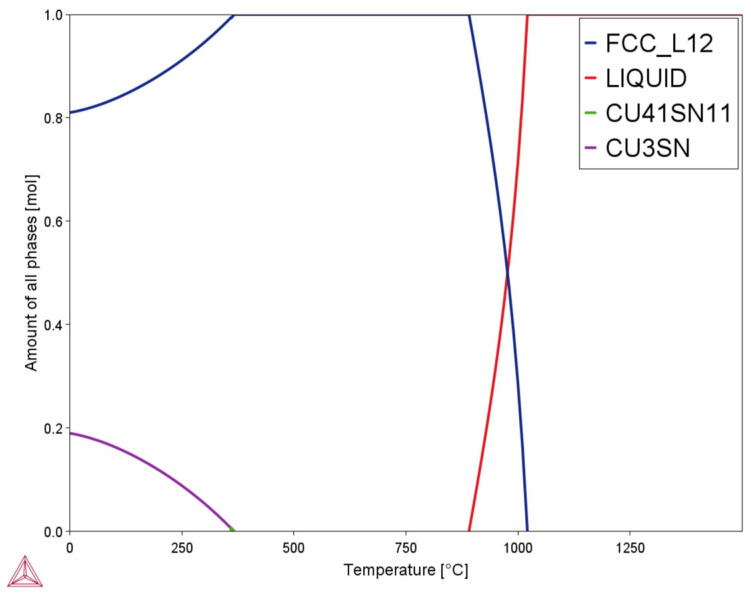
Thermo-Calc one-axis graph predicting the four phase equilibria at varying temperatures for Cu-10Sn alloys.

To better understand the variation behavior of the hardness of the samples, thermodynamics modeling was performed. Figure 6 shows the one-axis calculation of phase diagrams obtained using Thermo-Calc™ 2023b software. The evolution of the amounts of stable phases at equilibrium for four different phases, namely FCC_L12, liquid, Cu41Sn11, and Cu3Sn, can be observed at varying temperature ranges. Based on the thermodynamics modeling results, when the temperature increased from room temperature to 300 °C, the Cu3Sn phase continuously decreased.

To quantitatively predict the specific amount of stable Cu3Sn phases present at each low-temperature heat treatment point based on the thermodynamics modeling results, single point calculation was performed. Table 2 depicts the reduction in mole fraction of stable Cu3Sn phases with increasing temperatures. This explains the hardness test results, wherein, as the heat treatment temperature exceeded 180 °C, the average hardness of the samples gradually decreased with increasing temperature. However, if one strictly adhered to the thermodynamics modeling results under equilibrium conditions (specifically, the amounts of the Cu3Sn phase in the samples), maximum hardness was expected in the sample heat-treated at 140 °C due to precipitation hardening, as it contained the most Cu3Sn secondary phase. Nevertheless, the sample heat treated at 140 °C exhibited lower hardness than the counterparts heat treated at 180 °C and 220 °C.

The discrepancies noted between the Thermo-Calc simulations and the experimentally tested hardness results for the low-temperature heat-treated samples were mainly due to the following reasons. Although the 140 °C heat-treated sample was anticipated to exhibit the highest hardness value among all the heat-treated samples, it is worth noting that the thermodynamics modeling results were based on equilibrium conditions. AF LPBF parts are widely accepted in non-equilibrium states due to the extremely fast cooling rate [49]. Holding the AF parts at a temperature above room temperature was beneficial for the transition from non-equilibrium to equilibrium states. Specifically, experimental observations indicated that maintaining a temperature above 180 °C for 60 min was effective in driving this transition process. As the heat treatment temperature increased from 180 °C to 300 °C, the content of the Cu3Sn phase gradually decreased, which accounted for the diminishing hardness test results. However, a temperature of 140 °C was insufficient to drive an effective non-equilibrium to equilibrium transition. Less Cu3Sn phase was generated at 140 °C, particularly when compared with counterparts treated at 180 °C and 220 °C, which explained the lower hardness value of the former. Meanwhile, it was also clear that all the heat-treated samples showed higher average hardness results, which was mainly due to the generation of Cu3Sn phase after heat treatment.

### 3.3. Thermal Diffusivity Evaluation

**Figure 7:**
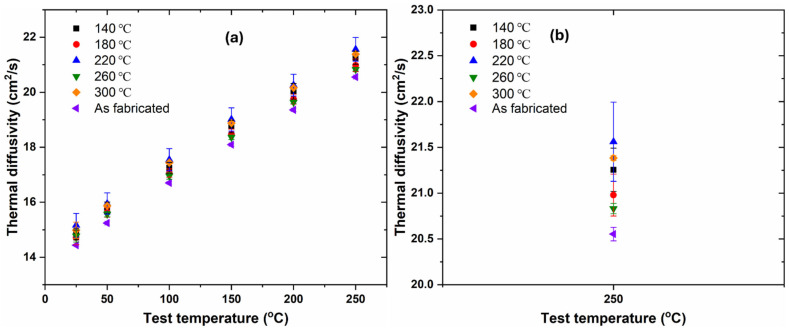
(**a**) Thermal diffusivity of both AF and heat-treated samples against test temperatures; (**b**) Thermal diffusivity of AF and heat-treated samples at 250 °C test temperature.

Figure 7a shows thermal diffusivity test results of both AF and all low-temperature heat-treated samples from room temperature to 250 °C. The error bars obtained results from averaging the thermal diffusivity values of repeated measurements. It was obvious that the trend in thermal diffusivity values for all samples was qualitatively similar across all test temperatures, i.e., thermal diffusivity values increased nearly linearly as temperature rose from 25 °C to 250 °C for both AF and low-temperature heat-treated samples, consistent with previous studies [3,5,50] using a similar laser flash method. Figure 7b shows the thermal diffusivity values of the samples at a larger magnification. It is clearly seen that the thermal diffusivity of the AF sample was lower than that of the heat-treated samples, while no clear difference can be observed among the heat-treated samples. Clearly, low-temperature heat-treated samples showed higher thermal diffusivity values than the AF sample across all test temperatures. This observation indicated the fact that the low-temperature heat treatments showed positive effects in improving thermophysical properties of AF Cu-10Sn alloy parts.

**Figure 8:**
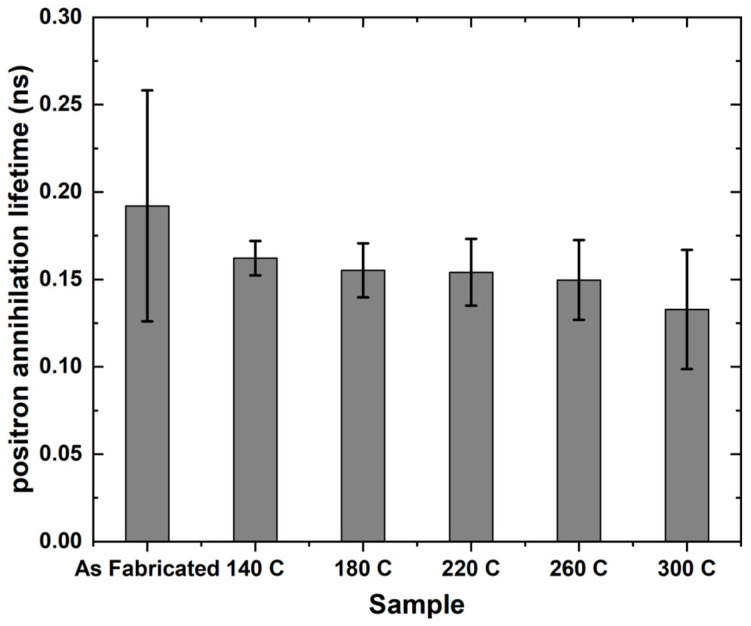
Positron lifetime results of AF and low-temperature heat-treated samples.

To discover the underlying mechanism, a PALS test was conducted. Figure 8 summarizes the lifetime results in nanoseconds (ns) for the AF and heat-treated samples using PALS. Clearly, based on Figure 8, it can be observed that the AF sample recorded the highest positron lifetime, averaging ~0.19 ns. The average positron lifetime then decreased with increasing heat treatment temperatures, with the 300 °C sample recording the lowest average lifetime of ~13 ns. The positron lifetime recorded for the present study predicted the concentration of atomic-level defects, specifically vacancies, present in the samples. The lower positron lifetime value indicated the lower concentration of atomic-level defects.

The reduction in the concentration of atomic-level defects induced by the LPBF process upon low-temperature heat treatment was expected. This reduction correlated well with their correspondingly low positron lifetimes. On the other hand, there was a presumption that the AF sample, with no post-heat treatment, still retained a high concentration of atomic-level defects. This was well correlated with the highest lifetime recorded among all other samples.

These speculations, based on the correlation between recorded positron lifetimes and concentration of atomic-level defects present, were well supported by the prior literature on the principle of operation of PALS and its application in defect detection and forensic investigation [40,51,52]. Therefore, the decrease in atomic-level defects after the low-temperature heat treatments was most likely the main reason why the thermal diffusivity of the samples increased.

**Figure 9:**
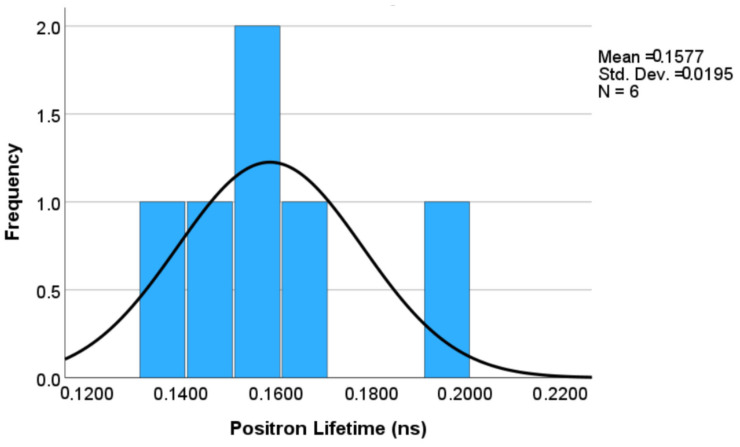
A positively skewed distribution curve of positron lifetime results among tested samples.

A statistical analysis of the positron lifetime data was conducted to examine the distribution of data and the correlation between the positron lifetime results of the AF samples and the heat-treated samples. Upon closer observation of Figure 9, the positron lifetime results showed a slight positive skewness. This positive skewness, quite expected, indicated that the positron lifetime values for most of the samples clustered around the mean value of 0.1577 ns, with a low standard deviation of 0.0195 ns. This was attributed to the results of the heat-treated samples predominantly ranging between 0.13 ns and 0.17 ns. However, there was a notable outlier in the positron lifetime data, which can be attributed to the AF sample with a high presence of atomic-level defects. However, careful observation of Figure 8 reveals that the error bar of the AF sample was quite large, indicating no guaranteed significant difference when considering average lifetimes and error bars together. To verify this, GraphPad Prism software (version 9.5.0) was also applied, and no significant difference was found. Therefore, more aspects should be considered.

As illustrated in the thermodynamics modeling results (Figure 6 and Table 2), it was discovered that more Cu3Sn (Sn-rich) phase was generated after heat treatments. The generation of the Cu3Sn phase further reduced the Sn content in the Cu matrix (α phase). As solid solute atoms were significant thermal barriers of heat conduction, the decreased content of Sn in the Cu matrix consequently increased the thermal diffusivity of the Cu-10Sn alloy. A detailed demonstration of how reducing Sn content in the Cu matrix (α phase) could increase the overall thermal diffusivity of Cu-10Sn alloy parts can be found in the authors’ previous study [5]. Therefore, the increase in thermal diffusivity of the heat-treated samples was most likely due to the combined effect of reduced atomic defect (i.e., vacancies) and reduced Sn solid solutes in the Cu matrix (α phase).

## 4. Conclusions

Cu-10Sn alloy samples were successfully fabricated via the LPBF-AM process. The effects of low-temperature heat treatments on mechanical and thermophysical properties of Cu-10Sn alloy samples and the evolution of defects were studied. The following inferences were drawn:

1. Both the AF and heat-treated samples consisted of a Cu-rich α phase and a Sn-rich δ phase with negligible Sn diffusion into the α-phase during fabrication and low-temperature heat treatment process based on XRD tests.
2. Both the AF and heat-treated samples exhibited similar grain structure and almost the same grain sizes. The value of the average grain size of the AF sample was ~2 μm, followed closely by an average size of ~1.9 μm across all heat-treated samples.
3. The heat-treated samples displayed higher average hardness compared with the AF sample. This finding was primarily attributed to the formation of Cu3Sn phases during low-temperature heat treatment, as predicted and verified through Thermo-Calc calculations. Peak hardness was achieved at a heat treatment temperature of 180 °C, reaching an average hardness value of 173.3 HV0.5.
4. The discrepancy between the hardness experimental test results and the Thermo-Calc calculations for low-temperature heat treatments at 140 °C suggested that a holding time of 60 min was not adequate to cause the formation of a higher concentration of Cu3Sn precipitates and consequently induce strengthening.
5. The thermal diffusivities of the heat-treated samples were slightly higher than that of the AF sample, which was mainly due to the reduction of atomic-level defects, vacancies, and reduced Sn content in Cu matrix after heat treatments.

## Figures and Tables

**Table 1:** Standard process parameters for the LPBF fabrication process.

Process Parameter	Value
Laser power	95 W
Scanning speed	200 mm/s
Hatch space	50 μm
Layer thickness	15 μm

**Table 2:** Single point Thermo-Calc simulation results of stable Cu3Sn phases at varying temperatures.

Temperature (°C)	Mole of Cu3Sn Phase (mol)
140	0.14731
180	0.12836
220	0.10664
260	0.08204
300	0.05427

## Data Availability

The raw data supporting the conclusions of this article will be made available by the authors, without undue reservation, to any qualified researcher. For additional in-formation on the datasets, please contact the corresponding author.
